# Analysis on evolution and research focus in psychiatry field

**DOI:** 10.1186/s12888-015-0482-1

**Published:** 2015-05-07

**Authors:** Ying Wu, Zhiguang Duan

**Affiliations:** School of Public Health, Shanxi Medical University, South Xinjian Road, Taiyuan, China

**Keywords:** Psychiatry, Evolution, Research focus, Visualization

## Abstract

**Background:**

With the dramatic rise in mental disorders and mental illnesses, psychiatry has become one of the fastest growing clinical medical disciplines. This has led to a rise in the number of scientific research papers being published in this field.

**Methods:**

We selected research papers in ten psychiatric journals that were published during 1983 to 2012. These ten journals were those with the top Impact Factor (IF) as indicated by the Science Citation Index Expanded (SCI-Expanded). We utilized information visualization software (CiteSpace) to conduct co-citation and Hierarchal clustering analysis to map knowledge domains to determine the evolution and the foci of research in this field.

**Results:**

In the evolution of the field of psychiatry, there were four stages identified. The result of hierarchal clustering analysis revealed that the research foci in the psychiatric field were primarily studies of child and adolescent psychiatry, diagnostic and classification criteria, brain imaging and molecular genetics.

**Conclusion:**

The results provide information about the evolution and the foci of the research in the field of psychiatry. This information can help researchers determine the direction of the research in the field of psychiatry; Moreover, this research provides reasonable suggestions to guide research in psychiatry field and provide scientific evidence to aid in the effective prevention and treatment of mental disorders.

## Background

Mental disorders pose a risk to the health of humans; in addition, they seriously affect individuals’ social lives. It is estimated that more than 4.5 billion persons suffer from mental illness worldwide [1]. According to the World Health Organization, it is estimated that mental illness is the top contributor to Disability-Adjusted Life Years (DALYs), which surpasses the contribution of cardiovascular disease, respiratory system disease, and malignant tumours [2-4]. With the dramatic rise in mental disorders and mental illness, psychiatry has become one of the fastest growing clinical medical disciplines.

The field of psychiatry was formed more than 100 years ago, and during that time frame it has grown considerably. The growth of the field was particularly apparent in later part of the 20th century; indeed, psychiatric research has made advances in methodology as well as in its application to the field of biomedicine. A large proportion of scientists have devoted their efforts to the field of psychiatry that has resulted in contributions to the extant literature. In the current study, we used visualization analysis to map the development of the field of psychiatry. Authors, published papers, and major themes in the field of psychiatry were identified to elucidate the direction of psychiatric research in order to prevent and cure mental illnesses.

## Methods

We selected 85,612 manuscripts from the ten psychiatric journals with the top Impact Factors (IF) from Science Citation Index Expanded (SCI-Expanded) from 1983 to 2012.These ten psychiatric journals were from JCR (Journal Citation Reports) in Web of Science in 2012 (see Table 1). This window of time was divided into three time periods: 1983–1992, 1993–2002, and 2003–2012. The date of each bibliographic record contained the title, author names, abstract, key words, and references, et al.

**Table 1:** **10 Representative journals in psychiatry field**

Rank	Journal title	Impact factor
1	Molecular Psychiatry	14.897
2	American Journal of Psychiatry	14.721
3	Archives of General Psychiatry	13.772
4	Biological Psychiatry	9.247
5	World Psychiatry	8.974
6	Neuropsychopharmacology	8.678
7	Schizophrenia Bull	8.486
8	Psychotherapy and Psychosomatics	7.230
9	Journal of the American Academy of Child and Adolescent Psychiatry	6.970
10	British Journal of Psychiatry	6.606

This study used document co-citation analysis which is a method based on the reference to study on the evolution and research focus in psychiatry field. In 1973, Small and Marshakora come up with the theory of document co-citation, that is when paper A and paper B are co-cited by paper C at the same time, the relationship between paper A and paper B is co-citation relationship [5,6]. After document co-citation analysis, the clusters in research fields can be received and the change of the clusters in every period can reflect evolution in research field. In order to intuitively display the our research results, we made knowledge mapping by the visualization software CiteSpace (http://cluster.cis.drexel.edu/~cchen/citespace), which is based on JAVA application that analyses the research focus in the field of psychiatry. CiteSpace was developed by Dr. Chaomei Chen of Drexel University in the United States. Dr. Chaomei Chen has studied on the method of information visualization and is an international expert in this field. In CiteSpace, we set the ‘time slicing’ to be 1983–1992, 1993–2002, and 2003–2012, and the ‘years per slice’ was set to ‘1’. The ‘team source’ selects were ‘title’, ‘abstract’, ‘author keywords’ and ‘keywords plus’. The ‘node types’ selects were ‘cited reference’. We set the ‘top *N* per slice’ to be ‘50’ which means that the 50 documents with the highest cited frequency were selected for each ‘time slicing’. In addition, we chose ‘pathfinder’ for the network analysis. The nodes and lines in the network were generated automatically [7]. There were different size and colour nodes that represented the articles that constructed the whole network. Citation tree-rings represented the citation history of an article. The colour of a citation ring indicated the time of a corresponding citation. The thickness of a ring was proportional to the number of citations in a given time slicing [8]. The purple ring represented the key document that was associated with an important theory and/or a new concept. Visualization instrument is a new generation of information visualization techniques that is adapted to multivariate, time-sharing, and dynamic complex network analysis. This method allows researchers to observe and understand information easily in order to identify a model and the regularities of citations behind a mass date [9]. Centrality reflects the status and rights of activities in their social network. In a collaborative network, if an entity has a high centrality, it is considered the “central entity” and possesses and controls a great deal of research resource in the collaborative network.

## Results and discussion

### Results of the evolution of research in psychiatry field From 1983 to 1992

**Figure 1:**
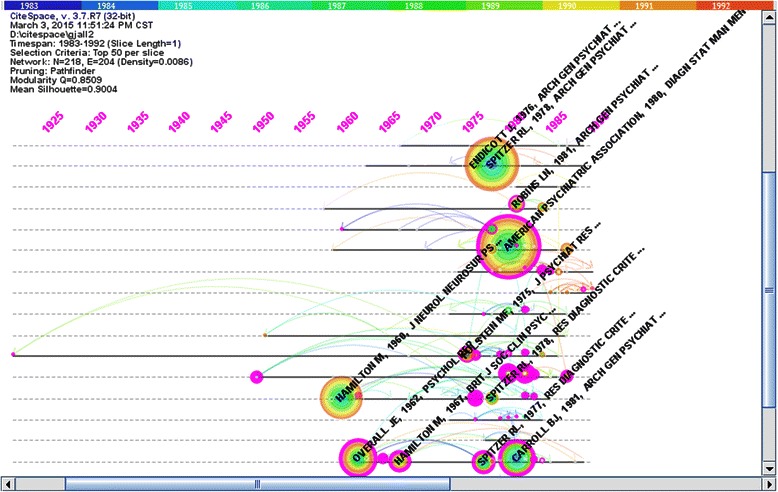
Co-citation network of documents (1983–1992). Figure 1 was the time-zone visual network of 218 co-cited articles in psychiatry field from 1983 to 1992 based on 10 one –year slices. The numbers at the top area represented years. There were total 44 purple rings which represented the articles’ centralities more than 0.1.The pivotal-points were identified with representative articles’ authors, publication years and journal titles.

First, we used CiteSpace to choose corresponding nodes to map the network of co-cited articles in the field of psychiatry from 1983 to 1992 (see Figure 1). There were a total of 218 nodes and 204 lines. Then, we selected the critical nodes which the centrality was more than 0.1 (see Table 2).

**Table 2:** **Information of main documents (1983–1992)**

Centrality	Document title	Journal title	Author	Publication year
0.75	Molecular pathology of schizophrenia-more than one disease process	British Medical Journal	Crow,TJ	1980
0.74	The influence of family and social factors on the course of psychiatric illness	Archives of General Psychiatry	Vaughn,CE	1976
0.72	Negative v positive Schizophrenia	Archives of General Psychiatry	Andreasen,NC	1982
0.58	Assessment of negative and positive symptoms in schizophrenia	Schizophrenia Bull	Lewine, RRJ	1983
0.55	Praecox dementia and schizophrenia	Science	Bleuler, E	1950
…	…	…	…	…
0.12	National institute of mental health diagnostic interview schedule	Archives of General Psychiatry	Robins, LN	1981
0.12	Cerebral glucography with positron tomography	Archives of General Psychiatry	Buchsbaum,MS	1982
0.12	Cortical secretion in relation to age in major depression	Psychosomatic Medicine	Asnis, GM	1981
0.11	Neuroendocrine regulation in depression	Archives of General Psychiatry	Carroll,BJ	1976
0.11	Temporal-lobe pathology in schizophrenia	American Journal of psychiatry	Suddath,RL	1989

As shown in Figure 1, the earliest appearance of an article published in the field of psychiatry was “Psychological work experiments” written by Kraepelin, E in 1921[10]. In this paper, ‘descriptive psychiatry’ was established, which laid the foundation for psychiatry; indeed, Kraepelin is regarded as the contemporary father of psychiatry. In 1950, *Praecox dementia and schizophrenia* was written by the famous psychiatrist Bleuler, E. In this paper, the clinical manifestation of schizophrenia was described in detail. In addition, the ‘4A’ symptoms of schizophrenia (associative disorder, affective disorder, ambivalent disorder, and autistic disorder) were introduced.

During this period of time, the field of psychiatry primarily focused on two research directions. One was the diagnosis of mental disorders. In 1962, “The brief psychiatric rating-scale” was compiled by Overall, JE [11]. This scale was adapted to functional psychosis and has been widely used in international collaborative research. In 1975, Spitzer, RL compiled the Diagnostic and Statistical Manual of Mental Disorders in the article titled “Clinical criteria for psychiatric diagnosis and DSM-III” [12]*.* The other focus of the field was psychopathology and the abnormal morphology of the brain. There were two key articles about psychopathology. The first was published in 1980 and was titled “Molecular pathology of schizophrenia-more than one disease process” by Crow, TJ [13]; the second was published in 1982 by Andreasen, NC and was titled “Negative V positive schizophrenia-definition and validation” [14]. The key articles about the abnormal morphology of the brain were “Cerebral ventricular size and neuropsychological impairment in young chronic schizophrenics” written by Golden, CJ in 1980 [15], and “Persistence of cerebral in chronic schizophrenia as determined by positron emission tomography” written by Wolkin, A in 1985 [16].

Moreover, there were three additional research directions during this time period. First, the paper titled “The diagnosis of depressive syndromes and the prediction of ECT response” by Carney, MWP in 1965 discussed the clinical efficacy and securing of ECT in depressive patients [17]. Second, the article titled “The influence of family and social factors on the course of psychiatric illness. A comparison of schizophrenic and depressed neurotic patients” written by Vaugh, CE in 1976 analyzed the societal and familial factors that influenced mentally ill patients [18]. Third, the paper written by Carroll, BJ titled “A specific laboratory test for the diagnosis of melancholia” provided evidence validating the use of dexamethasone suppression test (DST) for the diagnosis of melancholia [19].

### From 1993 to 2002

**Figure 2:**
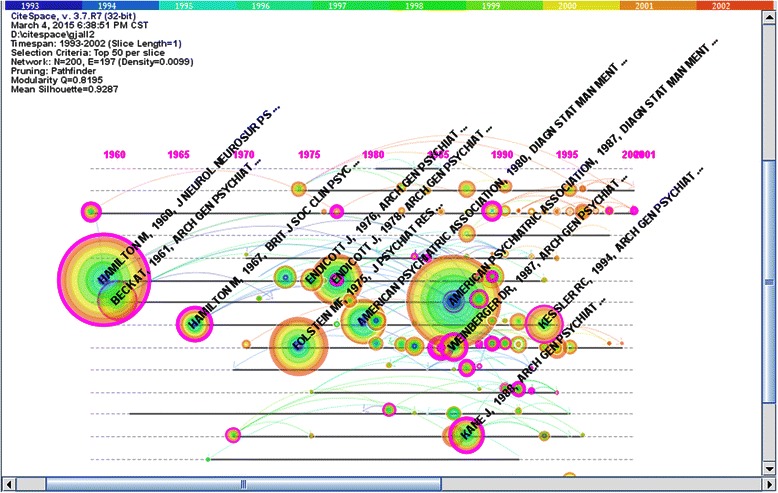
Co-citation network of documents (1993–2002). Figure 2 was the time-zone visual network of 200 co-cited articles in psychiatry field from 1993 to 2002 based on 10 one –year slices. The numbers at the top area represented years. There were total 41 purple rings which represented the articles’ centralities more than 0.1.The pivotal-points were identified with representative articles’ authors, publication years and journal titles.

Next, we used CiteSpace to select the corresponding nodes to map the network of co-cited articles in the field of psychiatry from 1993 to 2002 (see Figure 2). There were a total of 200 nodes and 197 lines. We then chose the critical nodes which the centrality was more than 0.1 (see Table 3).

**Table 3:** **Information of main documents (1993–2002)**

Centrality	Document title	Journal title	Author	Publication year
0.46	Neuropsychological assessment of monozygotic twins discordant for schizophrenia	Archives of General Psychiatry	Goldberg, TE	1990
0.45	Physiologic dysfunction of dorsolateral prefrontal cortex in schizophrenia	Archives of General Psychiatry	Weinberger,DR	1986
0.38	Auditory hallucinations and smaller superior temporal gyral volume in schizophrenia	American Journal of Psychiatry	Barta,PE	1990
0.38	Serotonergic studies in patients with affective and personality disorders	Archives of General Psychiatry	Caccaro,EF	1989
0.37	A diagnostic interview-the schedule for affective disorders and schizophrenia	Archives of General Psychiatry	Spitzer,RL	1978
…	…	…	…	…
0.14	Strong inference ,theory testing, and the neuroanatomy of schizophrenia	Archives of General Psychiatry	Carpenter,WT	1993
0.13	Reciprocal limbic-cortical function and negative mood: converging PET findings in depression and normal sadness	American Journal of Psychiatry	Mayberg, HS	1999
0.11	Negative symptoms in schizophrenia	Archives of General Psychiatry	Andreasen,NC	1982
0.11	A neurohistological correlate of schizophrenia	Biological Psychiatry	Kovelman, JA	1984
0.1	Abnormalities of the left temporal lobe and thought disorder in schizophrenia: a quantitative magnetic resonance imaging study	New England Journal of Medicine	Shenton,ME	1992

As depicted in Figure 2, the earliest appearance of a key article during this time period was in 1959. Specifically, the paper titled “The assessment of anxiety states by rating” written by Hamilton, M in 1959 was a report about the Hamilton Anxiety Scale (HAMA); this was a widely used assessment of anxiety [20]. In 1960, Hamilton, M also published “A rating scale for depression”*,* which was a widely used depression scale [21]. Then, in 1967, Hamilton, M published the article titled “Development of a rating scale for primary depressive illness”, which was also a key document during this period of time [22]. Furthermore, the article: “A rating scale for extrapyramidal side effects” published by Simpson, GM in 1970 discussed the extrapyramidal side effects of the psychotropic drug scale [23]. Moreover, in 1978, Spitzer, RL published the paper “A diagnostic interview: The schedule for affective disorders and schizophrenia”; this measure (the Schedule for Affective Disorders and Schizophrenia (SADS)) was developed to reduce information variance in both the descriptive and diagnostic evaluation of a subject [24].

Psychopathology and abnormal morphology of the brain was also a main research direction during this period of time, and the representative publications increased significantly. In 1982, “Negative symptoms in schizophrenia-definition and reliability” was written by Andreasen, NC; this paper described the negative symptoms of schizophrenia [14]. In 1984, Kovelman, JA wrote “A neurohistological correlate of schizophrenia”. This paper elaborated on the relationship between the number of pyramidal cell in the brain and schizophrenia [25]. In 1985, “Basal ganglia and limbic system pathology in schizophrenia” was written by Bogerts, B and elaborated on the relationship between the basal ganglia, limbic system and schizophrenia [26]. Weinberger, DR published “Physiologic dysfunction of dorsolateral prefrontal cortex in schizophrenia” [27] and “Implications of normal brain development for the pathogenesis of schizophrenia” [28] in 1986 and 1987, respectively. These two documents suggested that the relationship between the dorsolateral prefrontal cortex and schizophrenia was linked to regionally specific cognitive function, and was not a nonspecific epiphenomenon*.* In the research on psychopathology during this time, “The positive and negative syndrome scale (PANSS) for schizophrenia” was written in 1987 by Kay, SR; this well-known scale was used to distinguish type schizophrenia from type schizophrenia, with the former based on positive syndromes and the latter on negative syndromes [29].

In addition, there were several new key articles that indicated new changes in the field during this period of time. For instance, there was research on antipsychotic drugs; in 1988, “Clozapine for the treatment-resistant schizophrenic” was written by Kane, J. This article described the treatment of schizophrenia with clozapine [30]. In addition, research on neurological biochemistry emerged. For instance, in 1989, Coccaro, EF wrote “Serotonergic studies in patients with affective and personality disorders”. This study indicated that a reduction in central serotonergic functioning was present in a subgroup of patients with major affective and personality disorders, and was also correlated with suicidal behaviours and impulsive aggressive behaviour. Furthermore, research on brain imaging also appeared [31]. In 1992, “Abnormalities of the temporal lobe and thought disorder in schizophrenia: A quantitative magnetic-resonance-imaging study” was written by Shenton, ME. This study described that schizophrenia involved localized reductions in gray matter in the left temporal lobe, and that this was related to the degree of thought disorders in patients; this was revealed via studies that utilized the following methodologies: post-mortem examinations, CT scans, and magnetic resonance imaging (MRI) [32]. The other key article during this time was “Reciprocal limbic-cortical function and negative mood: Converging PET findings in depression and normal sadness”, which was written by Mayberg HS in 1999. In this article, the relationship between reciprocal limbic-cortical functioning and negative mood in depression was found via PET scans [33].

### From 2003 to 2012

**Figure 3:**
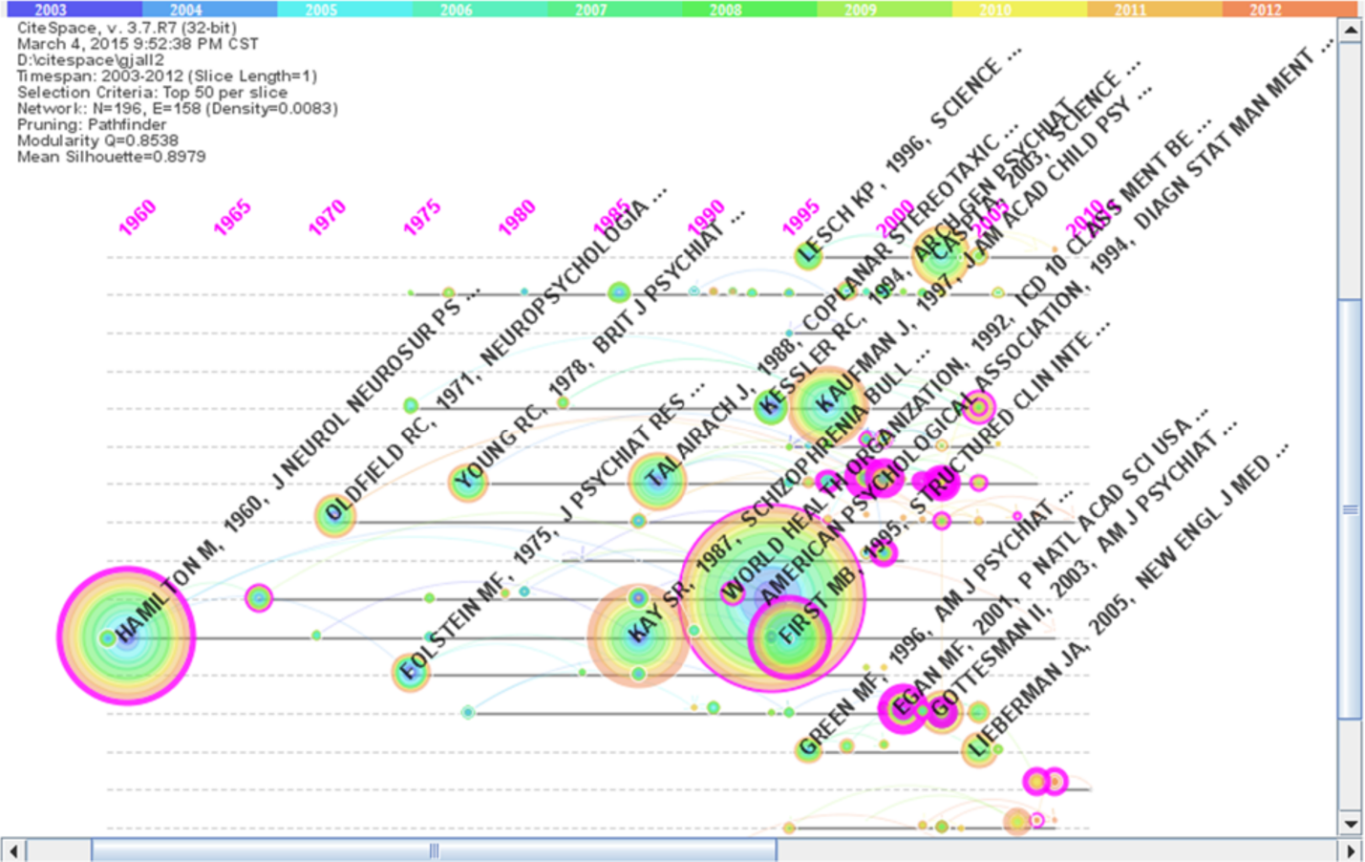
Co-citation network of documents (2003–2012). Figure 3 was the time-zone visual network of 196 co-cited articles in psychiatry field from 2003 to 2012 based on 10 one –year slices. The numbers at the top area represented years. There were total 33 purple rings which represented the articles’ centralities more than 0.1.The pivotal-points were identified with representative articles’ authors, publication years and journal titles.

CiteSpace was used to select corresponding nodes to map the network of co-cited articles in psychiatry field from 2003 to 2012 (see Figure 3). There were a total of 196 nodes and 158 lines. The critical nodes were chosen which the centrality was more than 0.1 (see Table 4).

**Table 4:** **Information of main documents (2003–2012)**

Centrality	Document title	Journal title	Author	Publication year
0.71	Neurobiology of emotion perceptionI:the neural basis of normal emotion perception	Biological Psychiatry	Phillip, ML	2003
0.64	Reciprocal limbic-cortical function and Negative mood: converging PET finding in depression and normal sadness	American Journal of Psychiatry	Mayberg, HS	1999
0.48	Effect of COMT Val(108/158)Met genotype on frontal lobe function and risk for schizophrenia	Proceedings of the NationalAcademy of Sciences of theUnited States of America	Egan,MF	2001
0.48	Emotion circuits in the Brain	Annual Review of Neuroscience	Ledoux, JE	2000
0.48	Founctional neuroanatomy of Emotion: A Meta-Analysis of Emotion Activation Studies in PET and fMRI	Neurolmage	Phillip, ML	2002
…	…	…	…	…
0.13	Prevalence, severity and comorbidity of 12-month DSM-IV disorders in the national comorbidity survey replication	Archives of General Psychiatry	Kessler, RC	2005
0.13	Disruption of two novel genes by a translocation co-segregating with schizophrenia	Human Molecular Genetics	Millar, JK	2000
0.11	Lifetime prevalence and age-of-onset distribution’s of DSM-IV disorders in the national comorbidity surrey replication	Archives of General Psychiatry	Kessler, RC	2005
0.11	Neuregulin 1 and susceptibility to schizophrenia	American Journal of Medical Genetics	Stefansson, H	2002
0.1	Meta-analysis of whole-genome linkage scans of bipolar disorder and schizophrenia	Molecular Psychiatry	Badner JA	2002

As shown in Figure 3, the earliest appearance of key articles was in 1960 and 1967. These articles were titled "A rating scale for depression" and "Development of rating scale for primary depressive illness", both written by Hamilton, M. The diagnosis of mental disorders was a classic research direction that has appeared in these three time periods. However, we could not detect key documents during the end of the 1960s to the middle of the 1990s, which indicated that classical documents in the field of psychiatry were gradually decreasing and traditional research directions were gradually disappearing.

Emotion became the new research direction in psychiatry. In 2000, “Emotion circuits in the brain” was written by LeDoux, JE; this article proposed the notion that emotion, which influences perception, attention, memory and reading, was regarded as an important exciter and organizer of cognition, behaviour, social communication and development [34]. In 2002, Phan, KL published “Functional neuroanatomy of emotion: A Meta-analysis of emotion activation studies in PET and fMRI in Neuroimage”. This paper provided a critical comparison of findings across individual studies, and suggested that separate brain regions were involved in different aspects of emotion [35]. In 2003, “Neurobiology of emotion perception: The neural basis of normal emotion perception” was written by Phillips, ML. and proposed that the production of an affective state may depend on the level of activity within the ventral and dorsal system [36].

Moreover, brain imaging was also a research direction during this period of time. For instance, Mayberg, HS published the article: “Deep brain stimulation for treatment-resistant depression”; this study suggested that disrupting focal pathological activity in limbic-cortical circuits via electrical stimulation of the subgenual cingulated white matter effectively reversed symptoms in otherwise treatment-resistant depression [37]. In 2007, “Regional gray matter volume abnormalities in the at-risk mental state” was published by Borgwardt, SJ and suggested that individuals with an at-risk mental state (ARMS) had reductions in gray matter volume in areas of the brain [38].

In the 21st century, molecular genetics became the main research direction in the field of psychiatry. For instance, in 2001, Egan, MF published “Effect of COMT Val (108/158) Met genotype on frontal lobe function and risk for schizophrenia”. This study suggested that a relationship existed between a common functional polymorphism (Val 108/158 Met) in the Catechol-O-methyltransferase (COMT) gene and schizophrenia [39]. Moreover, Walsh, T wrote “Rare structural variants disrupt multiple genes in neurodevelopmental pathways in schizophrenia” in 2008. In this study, it was suggested that multiple, individually rare gene mutations altered neurodevelopmental pathways and contributed to the onset of schizophrenia [40]. Furthermore, in 2009, “Common polygenic variation contributes to risk of schizophrenia and bipolar disorder” was written by Purcell, SM. In this paper, evidence was provided for a substantial polygenic component in the risk of schizophrenia; this risk was identified via molecular genetics and involved thousands of common alleles of very small effect [41].

### Results of the identification of research foci in psychiatry field From 1983 to 1992

**Figure 4:**
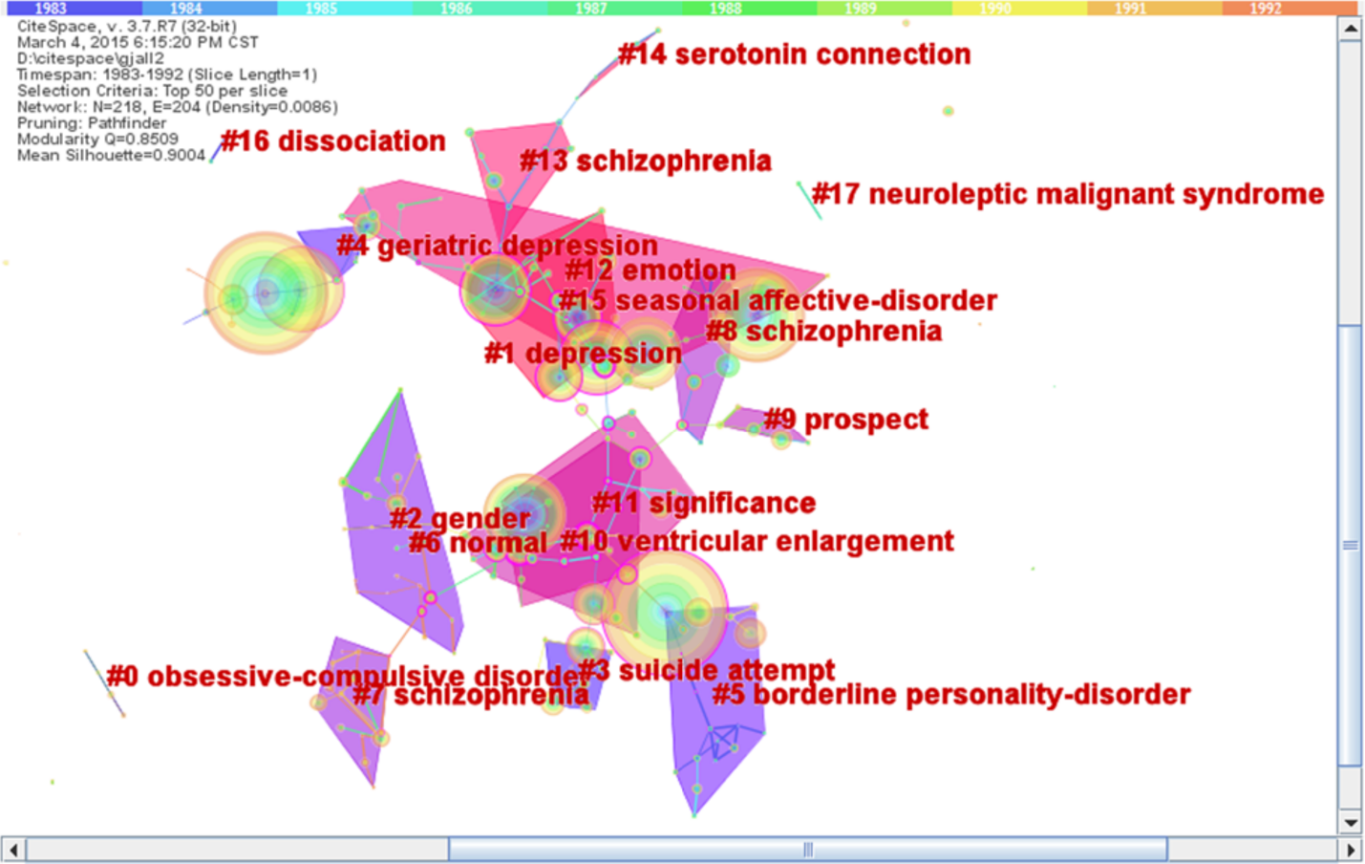
Research focus in psychiatry field (1983–1992) Figure 4 was time-zone visual clustering network of 218 co-cited articles in psychiatry field from 1983 to 1992 based on 10 one-year slices. There were totally 18 sub-networks. The sequence number of sub-networks and cluster top terms were marked in the central area.

In order to further detect the foci of research in the field of psychiatry, we mapped hierarchal clustering network of co-cited articles (see Figure 4). The analysis indicated 18 sub-networks (see Table 5). We selected the log-likelihood ratio (LLR) and extracted the terms to identify each cluster. We inferred six research trends according to the cluster terms and research directions.

**Table 5:** **Information of clustering sub-network (1983–1992)**

Sub-network	Number of document	Representative document	Cluster top term	Research focus
0	4	Local cerebral glucose metabolic rates in obsessive-compulsive disorder	Obsessive-compulsive disorder	Obsessive-compulsive disorder
1	30	Research diagnostic criteria-rationale and reliability	Depression	Depression
2	4	Sex-differences in schizophrenia-timing or subtypes	Gender	Schizophrenia
3	9	National institute of mental health diagnostic interview schedule its history, characteristics and validity	Suicide attempt	Diagnosis of mental disorders
4	5	Social origins of depression-reply	Geriatric depression	Depression
5	18	The validity of DSM-III borderline personality disorder	Borderline personality-disorder	Diagnosis of mental disorders
6	19	Physiologic dysfunction of dorsolateral prefrontal cortex	Normal	Schizophrenia
7	14	Basal ganglia and limbic system pathology in schizophrenia-a morphometric study of brain volume and shrinkage	Schizophrenia	Schizophrenia
8	12	Family history method using diagnostic criteria reliability and validity	Schizophrenia	Schizophrenia
9	6	Protein measurement with the Folin Phenol Reagent	Prospect	Neurological biochemistry
10	14	Cerebral ventricular size and cognitive impairment in chronic schizophrenia	Ventricular enlargement	Schizophrenia
11	22	Diagnostic criteria for use in psychiatric research	Significance	Diagnosis of mental disorders
12	25	A rating scale for depression	Emotion	Diagnosis of mental disorders
13	9	Dexamethasone suppression test for melancholia	Depression	Depression
14	5	Bulimia treated with imipramine: a placebo –controlled, double-blind study	Serotonin connection	Neurological biochemistry
15	18	The brief psychiatric rating-scale	Seasonal affective-disorder	Diagnosis of mental disorder
16	2	Carbamazepin in manic –depressive illness: a new treatment	Dissociation	Drug treatment of mental disorders
17	2	Neuroleptic malignant syndrome	Neuroleptic malignant syndrome	Drug treatment of mental disorders

The first research trend was obsessive-compulsive disorder in sub-network 0. The representative document in this sub-network was “Local cerebral glucose metabolic rates in obsessive-compulsive disorder: A comparison with rates in unipolar depression and in normal controls” by Baxter, LR that was published in 1987. This study suggested that there were abnormalities in the local cerebral metabolic rates for glucose in the brain structures of individuals with obsessive-compulsive disorder [42].

The second research trend was depression and was identified in sub-network 1, 4, and 13. The representative article was “Research diagnostic criteria: Rationale and reliability” by Spitzer, RL; this paper was published in 1978. This study indicated that there was a high reliability for diagnostic judgments made using Research Diagnostic Criteria (RDC), which was a widely used method; this method was often used in studies of genetics, psychobiology of selected mental disorders and treatment outcomes [43].

The third research trend was the study of schizophrenia in sub-networks 2, 6, 7, 8 and 10. The representative manuscript was “Physiologic dysfunction of dorsolateral prefrontal cortex in schizophrenia”, which was published in 1986 and written by Weinberger, DR. The results of the study suggested that physiologic dysfunction of the dorsolateral prefrontal cortex was linked to cognitive functioning in patients with chronic schizophrenia [27].

The fourth research trend was the classification and diagnosis of mental disorders in sub-networks 3, 5, 11, 12 and 15. The representative document was “A rating scale for depression” written by Hamiliton, M in 1960. This paper described the well-known Hamilton Depression Scale (HAMD), which was the depression scale widely used in clinical applications [21].

The fifth research trend was neurological biochemistry in sub-networks 9 and 14. The representative publication was “Protein measurement with the Folin Phenol Reagent”written by Lowry, OH in 1951. In this paper, the theoretical basis of Lowry protein measurement was described; this method is used worldwide [44].

The sixth research trend was the treatment of mental disorders with drugs in the sub-networks 16 and 17. “Carbamazepin in manic–depressive illness: A new treatment” written by Ballenger, JC in 1980 was the representative paper. This paper indicated that Carbamazepine was a useful treatment for affective illness [45].

### From 1993 to 2002

**Figure 5:**
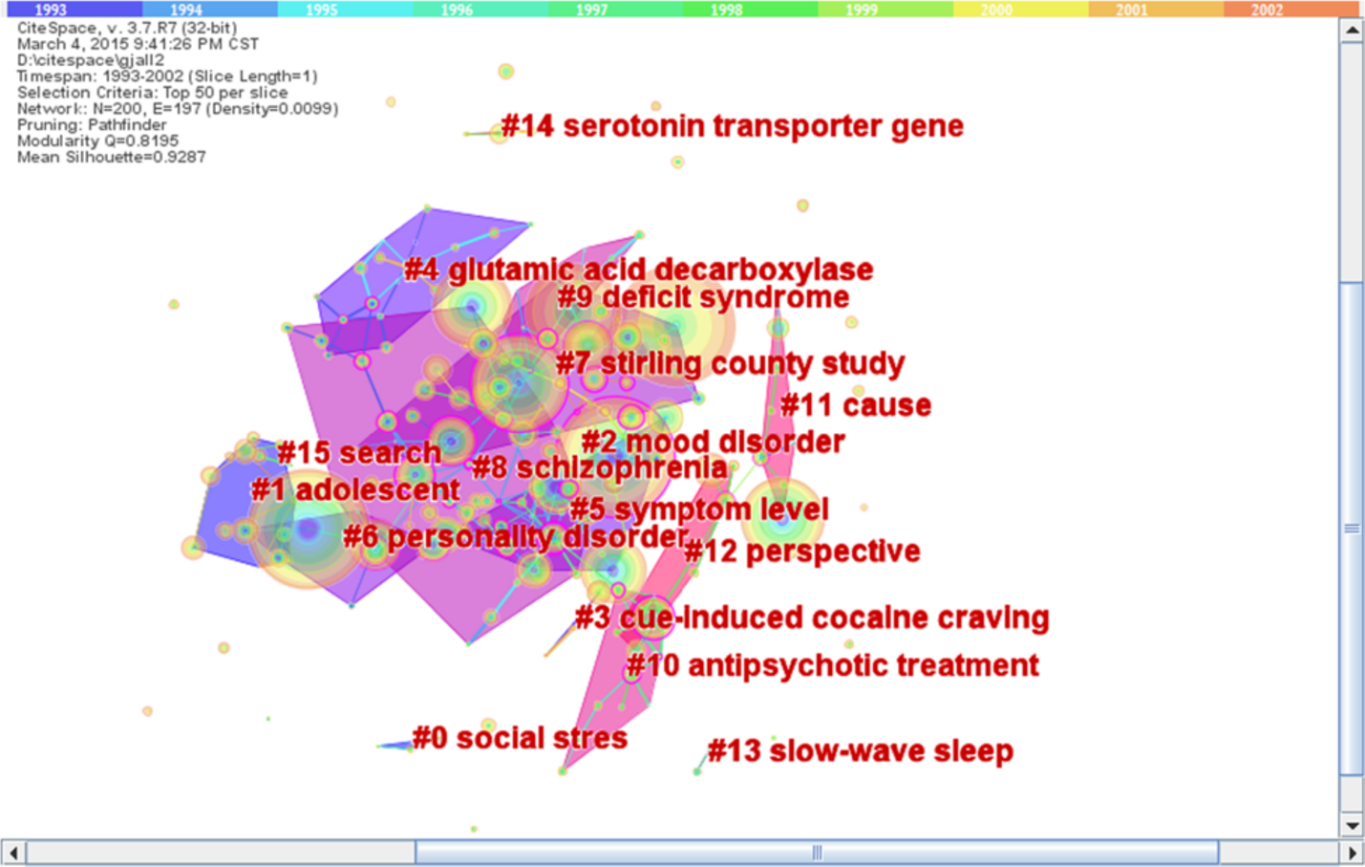
Research focus in psychiatry field (1993–2002). Figure 5 was time-zone visual clustering network of 200 co-cited articles in psychiatry field from 1993 to 2002 based on 10 one-year slices. There were totally 16 sub-networks. The sequence number of sub-networks and cluster top terms were marked in the central area.

To detect the research foci, we mapped the hierarchal clustering network of the co-cited documents (see Figure 5) and we gained 16 sub-networks (see Table 6). We selected the log-likelihood ratio (LLR) and extracted the terms to identify each cluster. According to the cluster term and research direction, we inferred eight research trends.

**Table 6:** **Information of clustering sub-network (1993–2002)**

Sub-network	Number of document	Reprehensive document	Cluster top term	Research focus
0	3	Elevated concentrations of CSF corticotrophin releasing factor like immuoreactivity in depressed-patients	Social stress	Depression
1	10	The NIMH diagnostic interview schedule for children Version 2.3(DISC-2.3): Description, acceptability, prevalence rates and performance in the MECA study	Adolescent	Mental disorders in childhood and adolescent
2	36	The assessment of anxiety –states by rating	Mood disorder	Diagnostic criteria of mental disorders
3	3	Activation of memory circuits during cue- elicited cocaine craving.	Cue-induced cocaine craving	Mental disorders associated with psychoactive substance abuse
4	17	Deficits in small interneurous in prefrontal and cingulated cortices of schizophrenic and schizoaffective patients	Glutamic acid decarboxylase	Schizophrenia
5	15	A rating scale for depression	Symptom level	Depression
6	10	An Inventory for measuring depression	Personality disorder	Depression
7	18	A comparison of diagnostic interview for depression in the stirling county study: challenge for psychiatric epidemiology	Stirling county study	Depression
8	44	Min-mental state practical method for grading for cognitive state of patients for clinician	Schizophrenia	Schizophrenia
9	10	The symptoms of chronic schizophrenia. a re-examination of the positive	Deficit syndrome	Schizophrenia
10	11	Positron emission tomographic analysis of central D1-Dopamine and D2-dopamine receptor occupancy in patients treated with classical neuroleptics and clozapine relation to extrapyramidal side effects	Antipsychotic treatment	Drug treatment of mental disorders.
11	8	The Brief Psychiatric Rating-scale	Cause	Diagnostic criteria of mental disorders
12	8	Clozapine for the treatment resistant schizophrenic-a double –blind comparison with chlorpromazine	Perspective	Drug treatment of mental disorders.
13	2	A manual of standardized terminology techniques and scoring system for sleep stages of Human subjects USA National Institute Health Publication 204 book	Slow-wave sleep	Sleep disorders.
14	3	Association of anxiety –related traits with a polymorphism in the serotonin transporter gene regulatory region	Serotonin transport gene	Genetic psychiatry
15	2	The future of genetic studies of complex human diseases	Search	Genetic psychiatry

The first research trend was depression in the sub-networks of 0, 5, 6, and 7. The representative document was “A comparison of diagnostic interview for depression in the stirling county study: challenge for psychiatric epidemiology” written by Murphy, JM. In this study, clinician-administered interviews corroborated disorders identified in lay-administered interviews for psychiatric epidemiology [46].

The second research trend was mental disorders in childhood and adolescence in sub-network 1. The representative paper was written by Shaffer, D and was titled “The NIMH Diagnostic Interview Schedule for Children Version 2.3(DISC-2.3): Description, acceptability, prevalence rates and performance in the MECA study”. This paper provided information about the diagnostic criteria of child and adolescent mental disorders [47].

The third research trend was the diagnostic criteria of mental disorders in sub-networks 2 and 11. The representative publication was “The assessment of anxiety states by rating” and was written by Hamilton, M and published in 1959. This paper described the well-known Hamilton Anxiety Scale (HAMA), which is widely used to assess anxiety states [20].

The fourth research trend was mental disorders associated with psychoactive substance abuse in sub-network 3. The representative publication was titled “Activation of memory circuits during cue-elicited cocaine craving” written by Grant,S in1996. This paper provided evidence of correlations between the cerebellum and self–reports of craving. These findings suggested a distributed neural network existed that integrated emotional and cognitive aspects of memory and linked environmental cues with cocaine craving [48].

The fifth research trend was the study of schizophrenia and located in sub-networks 4, 8 and 9. The representative document was written by Folstein, MF in 1975 and titled “Mini-mental state: A practical method for grading the cognitive state of patients for the clinician”. This document provided an assessment of grading cognitive states in schizophrenic patients [49].

The sixth research trend was the drug treatment of mental disorders in sub-networks 10 and 12. The representative paper was “Positron emission tomographic analysis of central D1-Dopamine and D2-dopamine receptor occupancy in patients treated with classical neuroleptics and clozapine relation to extrapyramidal side effects”; this paper was written by Farde L in 1992. This study suggested that the D1 occupancy was induced by clozapine and that flupentixol may contribute to the antipsychotic effects of these drugs [50].

The seventh research trend was sleep disorders in sub-network 13. The representative document in this sub-network was “A manual of standardized terminology techniques and scoring system for sleep stages of human subjects USA National Institute Health Publication 204 book”. This publication was written by Rechts CA in 1968 and set the standard for sleep stages [51].

The eighth research trend was the area of genetic psychiatry in sub-networks 14 and 15. The representative document was the “Association of anxiety–related traits with a polymorphism in the serotonin transporter gene regulatory region” written by Lesch KP in 1996. This study suggested that 5-hydroxytryptamine transporter (5-HTT) gene polymorphism was related to an anxious personality [52].

### From 2003 to 2012

**Figure 6:**
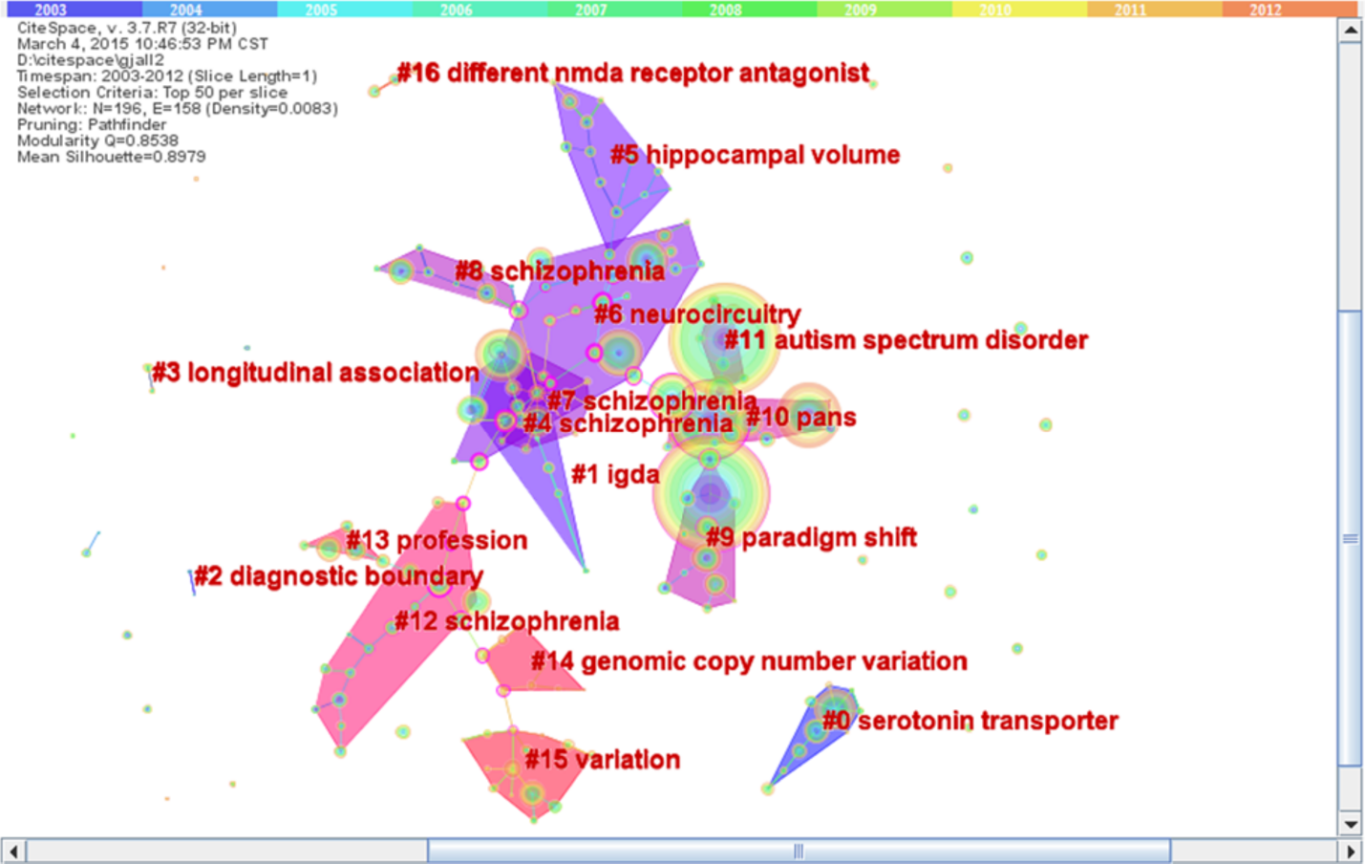
Research focus in psychiatry field (2003–2012). Figure 6 was time-zone visual clustering network of 196 co-cited articles in psychiatry field from 2003 to 2012 based on 10 one-year slices. There were totally 16 sub-networks. The sequence number of sub-networks and cluster top terms were marked in the central area.

We mapped hierarchal clustering network of co-cited documents to detect the research focus (see Figure 6) and gained 16 sub-networks (see Table 7). We selected the log-likelihood ratio (LLR) and extracted the terms to identify each cluster. We inferred six research trends based on the cluster terms and research directions.

**Table 7:** **Information of clustering sub-network (2003–2012)**

Sub-network	Number of document	Reprehensive document	Cluster top term	Research focus
0	10	Influence of lifestress on depression: moderation by a polymorphism in the 5-HTT gene	Serotonin transporter	Depression
1	35	Physical and genetic mapping of the IGDA locus at 6p25	IGDA	Genetic psychiatry
2	2	Mania-like symptoms suggestive of childhood-onset bipolar disorder in clinically referred children	Diagnostic boundary	Mental disorders of child and adolescent.
3	2	Prevalence and development of psychiatric disorders in childhood and adolescence	Longitudinal association	Mental disorders of child and adolescent.
4	10	Schedule for affective disorders and schizophrenia for school-age children present and life time version (K-SADS-PL):initial reliability and validity data	Schizophrenia	Mental disorders of child and adolescent.
5	14	Requirement of hippocampal neurogenesis for the behavioral effects of antidepressants	Hippocampal volume	Drug treatment of Mental disorders
6	23	Hemispheric lateralization of motor and speech functions after early brain lesion-study of 73 epileptic patients with intracarotid amytal test	Neurocircuitry	abnormal morphology of the brain
7	12	Implications of normal brain-development for the pathogenesis of schizophrenia	Schizophrenia	Schizophrenia.
8	9	A review of MRI findings in schizophrenia published in Schizophrenia Research	Schizophrenia	Schizophrenia.
9	12	New depression scale designed to be sensitive to change	Paradigm shift	Depression
10	13	A rating scale for depression	Pans	Depression
11	7	Min-mental state practical method for grading cognitive state of patients for clinician	Autism spectrum disorder	Schizophrenia.
12	17	The endophenotype concept in psychiatry: etymology and strategic intentions	Schizophrenia	Schizophrenia.
13	5	Effectiveness of antipsychotic drugs inpatients with chronic schizophrenia	Profession	Drug treatment of Mental disorders
14	8	Rare structural variants disrupt multiplegenes in neurodevelopmental pathways contribute to schizophrenia.	Genomic copy number	Genetic psychiatry
15	14	Haploview: analysis and visualization of LD and haplotype maps	Variation	Genetic psychiatry
16	3	The endophenotype concept in psychiatry: etymology and strategic intentions	Different NMDA receptor	Schizophrenia.

The first research trend was the study of depression in sub-networks 0, 9, and 10. The representative document was once again “A rating scale for depression” by Hamilton, M in 1960; this was the classic study of a well-regarded depression scale [21].

The second research trend was in the area of genetic psychiatry in sub-networks 1, 14, and 15. The representative publication was “Physical and genetic mapping of the IGDA locus at 6p25” written by Mears, AJ in 1997. This study introduced the relationship between IGDA locus at 6p25 and mental disorders [53].

The third research trend was the study of mental disorders in children and adolescents in sub-networks 2, 3 and 4. The representative publication was the “Schedule for affective disorders and schizophrenia for school-age children present and lifetime version (K-SADS-PL): Initial reliability and validity data” and was written by Kaufman, J in 1997. The results of this study suggested that the K-SADS-PL was a reliable and valid measure of childhood psychiatric diagnoses [54].

The fourth research trend was the drug treatment of mental disorders in sub-networks 5 and 13. The representative document was the “Requirement of hippocampal neurogenesis for the behavioral effects of antidepressants”*,* which was written by Santarelli, L in 2003. The findings of this study suggested that the behavioural effects of chronic antidepressants may be mediated by the stimulation of neurogenesis in the hippocampus [55].

The fifth research trend was the abnormal morphology of the brain in sub-network 6. The representative document was the article titled: “Hemispheric lateralization of motor and speech functions after early brain lesion-study of 73 epileptic patients with intracarotid amytal test”. This paper was written by Rey, M in 1988. This study introduced the relationships of those shifts to different variables related to the cerebral pathology [56].

The sixth research trend was the study of schizophrenia in sub-networks 7, 8, 11, 12 and 16. The representative document was the article titled “The endophenotype concept in psychiatry: Etymology and strategic intentions”, which was written by Gottesman in 2003. This study introduced the endophenotypes of schizophrenia with complex genetics [57].

## Conclusions

The study of psychiatry originated in the 19th century. In the 1920s, this field began to develop gradually. In 1921, Kraepelin, E published the study “Psychological work experiments”, which was the key document and laid the foundation for the study of psychiatry. As a result, Kraepelin was regarded as the contemporary father of psychiatry. In the mid and late 20th century, psychiatry entered into the field of biomedicine. In 1950, the famous psychiatrist Bleuler, E published the paper “Praecox dementia and schizophrenia”, which provided the ‘4A’ symptoms of schizophrenia. From the three periods of development and research directions from 1983 to 2012, we inferred four stages in the development of the field of psychiatry.

From the 1960s to the 1970s, the first stage of research about the scales of mental disorders. “The assessment of anxiety states by rating” and “A rating scale for depression”, both written by Hamilton, M were the representative document. Subsequently, Spitzer, RL continuously published documents about the scales of mental disorders, which played an important part in development of psychiatry.

The 1980s was the second stage of research on psychopathology and abnormal morphology of the brain. In 1980, “Molecular pathology of schizophrenia: More than one disease process” was written by Crow, TJ. In this paper, it was indicated that negative symptoms of schizophrenia represented a different process of illness or a different type of symptoms; this was an important advance in the study of developmental psychopathology in the psychiatric field. At this time, Weinberger, DR continuously published papers about the abnormal morphology of the brain in schizophrenia, which provided theoretical guidance for this area of study.

The 1990s was the third stage of research about the involvement of neurotransmitters in mental disorders. It was during this period of time that Coccaro, EF, Murray, AM and Egan, MF published papers which described the different neurotransmitters related to mental disorders; these papers opened up a new way of thinking for psychiatric research.

The early 21st century was the fourth stage of research; this period of time focused on emotion and molecular genetics in the field of psychiatry. Recently, with improvements in understanding as a result of the human genome project, genetic factors have been shown to play a key role in mental disorders; indeed, psychiatric research has gradually placed an emphasis on molecular genetics and the location of important candidate genes [58]. During this period of time, the papers written by Purcell, SM laid the foundation for research on molecular genetics in the field of psychiatry.

With mental disorders and mental illnesses dramatically rising, psychiatry has become one of the fastest growing disciplines of clinical medicine. Through hierarchal clustering, we found the research foci in the psychiatric field. During these three periods of time, the trends in psychiatry consistently focused on depression, schizophrenia, and drug treatments of mental disorders. The classification and diagnosis of mental disorders was also a research trend during the first and second period of time. As shown in Figures 4, 5, and 6, we found that obsessive-compulsive disorder and neurological biochemistry were research trends during the first period of time. However, during the second period of time, the research trends turned to sleep disorders.

In addition, in the last several years, mental disorders in childhood and adolescence, brain imaging and molecular genetics have been the focus of research in the psychiatric field. Due to the psychological and physiological characteristics during childhood and adolescence, the factors that affect adolescent mental health are different from those that affect adults. Therefore, the number of childhood and adolescent mental disorder patients has dramatically increased in recent years; thus, mental disorders in childhood and adolescence has become a research trend in this field. With the development of imaging technology, it is possible to examine the anatomic structure, function, and metabolic changes of the brain; thus, imaging technology is widely applied to the study of major mental disorders.

Furthermore, molecular genetics is one of the trends in psychiatry field today. Many studies have shown that mental disorders are complex polygenetic diseases; therefore, susceptibility genes of mental disorders were a focus of research in this field. With the great progress in theory and technology in molecular biology, research on genes is quickly developing. Furthermore, with the advancement of genome-wide association analysis, more candidate genes of mental disorders can be found. As mental disorders are bringing the biggest burden to the society, it is important to find genes that correspond to specific clinical symptoms of mental disorders. This will allow for the commencement of drug treatments according to the relevant genotypes. Moreover, this will lead to the personalized treatment for patients with different mental disorders [59,60].

Since the 1980s, the field of psychiatry has developed rapidly. The rapid development in several subjects, including neurological biochemistry, psychopharmacology, and molecular genetics, has allowed for the use of new techniques in brain imaging. In addition, the application of theories from psychology and sociology has also contributed to fundamental changes in the understanding of mental disorders; this has spurred the development of the biopsychosocial medical model. Presently, individuals can understand mental disorders at the molecular level as well as attend to the pathogenetic role in mental disorders of psychosocial factor. There will be great achievements in the field of psychiatry in the 21st century with the biopsychosocial perspective, modern medical theories, and new technical applications used to prevent, diagnose, and treat mental disorders.
